# CsPbI_3_ Perovskite Nanorods: Enhancing Fluorescence Efficiency and Environmental Stability via Trioctylphosphine Ligand Coordination

**DOI:** 10.3390/ma18071518

**Published:** 2025-03-28

**Authors:** Chengqi Liu, Zahir Abdalla, Xiaoqian Wang, Manrui Liu, Yanhui Jiao, Zisheng Tang, Qi Zhang, Yong Liu

**Affiliations:** State Key Laboratory of Advanced Technology for Materials Synthesis and Processing, International School of Materials Science and Engineering (ISMSE), Wuhan University of Technology, Wuhan 430070, China; liuchengqi42@163.com (C.L.); zahiralbashir3@gmail.com (Z.A.); 303568@whut.edu.cn (X.W.); liumanr14@163.com (M.L.); j1769877205@163.com (Y.J.); tangzs3076@163.com (Z.T.); zq13307239180@163.com (Q.Z.)

**Keywords:** 1D perovskite, nanorods, CsPbI_3_ perovskite, TOP, surface ligands

## Abstract

Metal halide perovskite nanorods hold great promise for optoelectronic applications. However, they tend to undergo phase transitions due to the instability of the crystal phase under environmental conditions, leading to a rapid decline in the fluorescence efficiency. Here, we report a method in which trioctylphosphine (TOP) directly serves as both the surface ligand and solvent to synthesize highly stable α-CsPbI_3_ nanorods (NRs). This approach produces monodisperse α-phase NRs with controlled sizes (1 μm and 150 nm in length, and an aspect ratio of 10:1), as confirmed by high-resolution transmission electron microscopy (TEM) and X-ray diffraction. The optimized NRs exhibit a high photoluminescence quantum yield of around 80%, as well as excellent environmental stability; after 15 days of storage, the photoluminescence quantum yield (PLQY) retention is 90%. Transient absorption spectroscopy shows that the carrier lifetime is extended to 23.95 ns and 27.86 ns, attributed to the dual role of TOP in defect passivation and hydrolysis suppression. This work provides a scalable paradigm for stabilizing metastable perovskite nanostructures through rational ligand selection, paving the way for durable perovskite-based optoelectronics.

## 1. Introduction

Metal halide perovskites (MHPs) are a class of materials known for their excellent optical properties and structural diversity, making them suitable for a wide range of optoelectronic applications [1,2,3,4,5,6]. The all-inorganic cesium lead iodide perovskite (CsPbI_3_) in its α-phase, with its nearly ideal bandgap (~1.73 eV), high carrier mobility (>100 cm^2^ V^−1^ s^−1^), and strong photoluminescence properties, holds significant potential for next-generation photovoltaic and nano-laser devices [2,7,8,9,10,11]. Among these materials, quasi-one-dimensional metal halide perovskite nanorods exhibit remarkable optical and electronic properties [12,13].

In recent years, research in this field has expanded rapidly, with significant progress made in understanding the growth mechanisms and exploring a wide range of potential applications [14,15,16,17,18,19]. However, the synthesis of CsPbI_3_ perovskite nanorods still faces considerable challenges, particularly in producing colloidal quantum dots with a high crystal quality and stability [20,21,22]. This is primarily due to the metastable nature of CsPbI_3_, which tends to undergo phase transitions from the black α-phase to the yellow phase under ambient temperature and humidity. These transitions result in the rapid degradation of optical activity, which remains a critical barrier to practical applications. In some CsPbI_3_ NR synthesis systems, significant challenges persist, such as non-radiative recombination induced by iodine vacancy defects on the surface of the one-dimensional structure. This significantly reduces the PLQY [23,24], with many reports indicating PLQY values below 50%. Furthermore, in non-vacuum environments, lattice hydrolysis accelerates phase transitions, causing a significant decay in PLQY within just seven days [25,26]. Currently, the primary approach researchers use to improve the stability of perovskites is by encapsulating perovskite nanocrystals, such as encapsulation in hydrophobic polymer matrices or borosilicate glass [27,28,29]. This method can effectively enhance the stability of perovskites. However, it not only increases the complexity of the fabrication process but also carries the potential risk that the encapsulating layer may affect the optical absorption properties of the perovskite material, thus posing certain limitations.

To address these challenges, this study developed a crystal-face-selective coordination strategy mediated by TOP, enabling the effective synthesis of nanorods with two different sizes (1 µm and 150 nm in length, with an aspect ratio of 10:1). The precise attachment of TOP to the surface of the perovskite nanorods was confirmed through energy-dispersive X-ray spectroscopy (EDS), X-ray photoelectron spectroscopy (XPS), and infrared spectroscopy. The TOP-modified α-CsPbI_3_ nanorods exhibited excellent optical performance and stability in ambient air, with an initial PLQY of 80%, retaining 90% of the initial PLQY after 15 days, significantly outperforming traditional ligand systems in terms of the performance degradation. Transient absorption spectroscopy measured average carrier lifetimes of 23.95 ns and 27.86 ns. This work provides a valuable reference for the precise synthesis and stability enhancement of perovskite nanostructures, with both theoretical and practical significance.

## 2. Materials and Methods

### 2.1. Materials

Cesium carbonate (Cs_2_CO_3_, 99.9%, Aladdin, Shanghai, China), oleic acid (OA, 80–90%, Aladdin, Shanghai China), lead (II) iodide (99.99% metals basis, Aladdin, Shanghai China), trioctylphosphine (TOP, 90%, Aladdin, Shanghai China), and n-hexane (Aladdin, Shanghai China, >99%) were used.

### 2.2. Methods

#### 2.2.1. Preparation of CsOA Precursors

A typical synthesis method involves adding 800 mg (2.46 mmol) of Cs_2_CO_3_ and 10 mL of OA into a three-necked flask. The system is then connected to a double-row tube and dried under vacuum at 120 °C for 1 h, ensuring the complete dissolution of the powder and resulting in a clear solution. The mixture was then cooled to 110 °C for later use.

#### 2.2.2. Synthesis of TOP-CsPbI_3_ NRs

**Figure 1:**
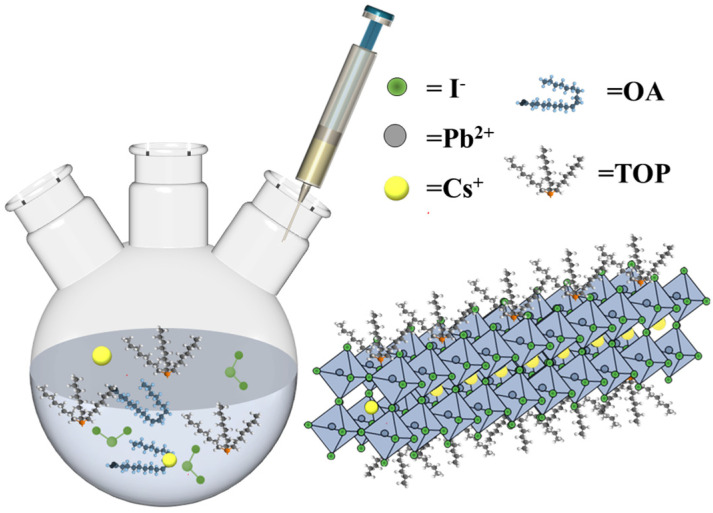
Flow diagram of the TOP-CsPbI_3_ NR synthesis process.

As shown in Figure 1, 200 mg of PbI_2_ and 10, 8, and 6 mL of TOP were added to another three-necked flask and dried under vacuum at 80 °C for 30 min. The system was then purged with nitrogen gas, heated to 120 °C under a nitrogen atmosphere, and maintained until completely dissolved.

The reaction temperature was then raised to 110 °C, and 0.2 mL of cesium oleate was quickly injected. The reaction was allowed to proceed for 30 s, during which the solution turned deep red. The reaction was quenched by placing the solution in an ice-water bath. The sample was then purified by centrifugation at 7000 rpm for 3 min, and the supernatant was removed. The residue was dispersed in n-hexane for further use.

### 2.3. Characterization

#### 2.3.1. Characterization of Material Morphology

TEM images were acquired using a JEOL JEM-1400 Plus transmission electron microscope (Tokyo, Japan) equipped with a thermionic electron gun at an accelerating voltage of 120 kV. HRTEM images were obtained on a Talos F200S-type field-emission transmission electron microscope (Hillsboro, OR, USA) at 200 kV. The size distribution of the NRs was determined by analyzing the TEM images via ImageJ software (Fiji).

#### 2.3.2. Structural and Surface Characterizations

X-ray Diffraction Analysis: XRD measurements were conducted using a Bruker D8 X-ray diffractometer, equipped with Cu-Kα radiation (λ = 1.54056 Å), and operating at 40 kV and 40 mA. Perovskite NR samples for the XRD analysis were prepared by drop-casting a diluted NR suspension in n-hexane onto a SiO_2_/Si substrate. X-ray Photoelectron Spectroscopy: XPS analysis was performed on a Thermo Scientific (Waltham, MA, USA) Kα XPS spectrometer, featuring a monochromatic Al-Kα X-ray source. The binding energy scale was calibrated using the C 1 s peak, with a binding energy of 284.8 eV, corresponding to the C–C bond. Fourier-Transform Infrared Spectroscopy: FTIR spectra were recorded using a Nicolet 6700 FTIR spectrometer (Waltham, MA, USA). For the analysis, concentrated NR solutions were drop-cast onto a clean glass substrate.

#### 2.3.3. Optical Characterization of Materials

The UV–vis absorption spectra were obtained using a Shimadzu UV-1800 spectrophotometer (Kyoto, Japan) with UVProbe 2.52 software. The steady-state photoluminescence (PL) spectra were measured on a Shimadzu RF-6000 spectrophotometer with LabSolutions RF software (Version 5.82). An excitation wavelength (λ_ex_) of 480 nm was used. Perovskite NR samples were prepared by diluting NR solutions in n-hexane within quartz cuvettes with a path length of 10 mm. The time-resolved photoluminescence spectra were measured at 685 nm using a 485 nm picosecond pulsed diode laser with a pulse width of ~200 ps and a pulse energy of 14 pJ (TRPL, Nano LED-C2 N-485L, HORIBA Scientific Company, Kyoto, Japan).

## 3. Results and Discussion

According to the existing literature, oleic acid and oleylamine are commonly used surface ligands in the synthesis of CsPbI_3_ perovskite. Studies have shown that organic ligands not only play a crucial role in the stability and surface modification of perovskites but also serve as key factors in the morphology control of CsPbX_3_ perovskites [30,31,32]. Specifically, by adjusting the type and concentration of ligands, precise control over the size, shape, and crystallinity of perovskite nanomaterials can be achieved. In this context, this study investigates the effect of using TOP as a surface ligand on the morphology of perovskite nanomaterials. The detailed experimental process is described in Section 2.

**Figure 2:**
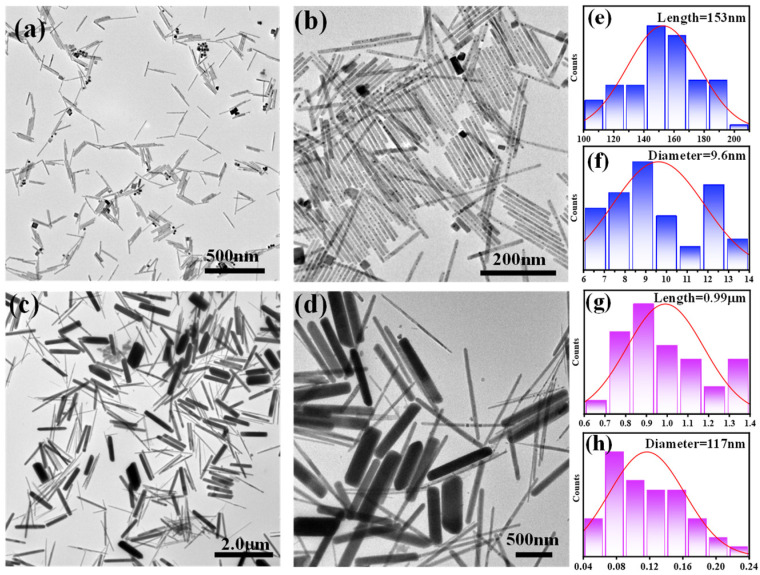
(**a**,**b**) TEM images of CsPbI_3_ nanorods synthesized at 10 mL of TOP. (**c**,**d**) TEM images of CsPbI_3_ nanorods synthesized at 8 mL of TOP. (**e**–**h**) The corresponding diameter and length distribution histograms.

By systematically controlling the concentration of the lead precursor (PbI_2_), we successfully achieved the size-controllable synthesis of CsPbI_3_ NRs. The TEM images (Figure 2a–d and Figure S1) reveal that the PbI_2_ concentration significantly affects the aspect ratio of the nanorods. Specifically, when the volume of the TOP solvent is 10 mL, the nanorods have an average length of 153 nm and a diameter of 9.6 nm (Figure 2e,f). However, when the TOP volume is reduced to 8 mL, the growth size of the nanorods increases substantially. This effect is particularly pronounced in high-concentration precursor solutions, where the crystal growth rate exceeds the nucleation rate. This suggests that, once the initial crystal nuclei form, they rapidly absorb the abundant surrounding reactants, leading to faster crystal growth. Consequently, as the reactant concentration increases, the size of the perovskite nanorods also enlarges, with the length and diameter growing to 0.99 μm and 117 nm, respectively (Figure 2g,h). As the size of the NRs increases, the specific surface area (the surface area to volume ratio) decreases with the increase in the volume. When a monolayer of TOP is maintained, it effectively reduces the total amount of surface ligands required to cover each unit of the CsPbI_3_ NRs. This experimental observation is directly associated with the reduction in the amount of TOP used.

However, under high-concentration conditions, the supersaturation of the reaction system increases significantly, which may lead to aggregation during the nucleation stage. Under the influence of van der Waals forces, the CsPbI_3_ nanorods tend to aggregate and entangle along the long axis, resulting in a non-uniform diameter distribution. Moreover, when the PbI_2_ concentration is further increased and the TOP volume is reduced to 6 mL, the size of the nanorods grows further to 1.87 μm (length) and 300 nm (diameter). This trend, where the size of the CsPbI_3_ nanorods increases as the TOP dosage decreases, is still observed. However, when the TOP dosage falls below 6 mL, the current experimental conditions may not be sufficient to effectively dissolve the perovskite nanorod precursors. Therefore, the TOP dosage range in this study was set between 6 mL and 10 mL. However, at this point, impurity byproducts such as nanocubes and nanosheets also appear in the reaction system (as shown in Figure S2).

**Figure 3:**
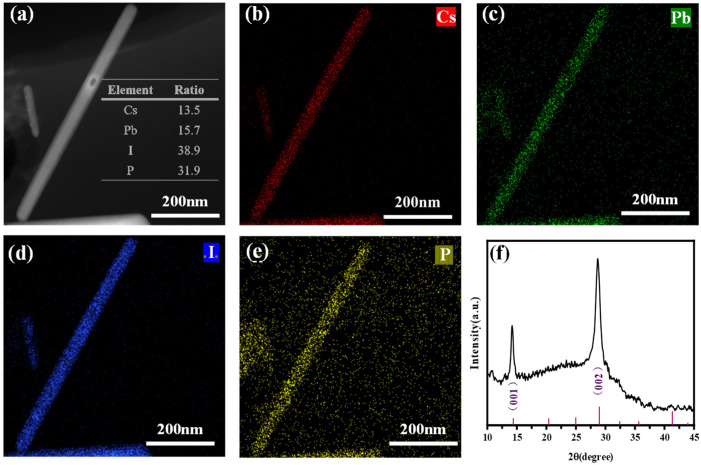
(**a**–**e**) High-resolution dark-field TEM image and elemental composition ratios. The corresponding elemental mapping images of Cs (**b**), Pb (**c**), I (**d**), and P (**e**). (**f**) XRD patterns of the CsPbI_3_ nanorods.

As shown in Figure 3a–e, we performed high-angle annular dark-field scanning transmission electron microscopy (HAADF-STEM) characterization on the CsPbI_3_ nanorods synthesized under 8 mL TOP conditions. Additionally, energy-dispersive X-ray spectroscopy (EDS) was employed to analyze the elemental distribution of Cs, Pb, and I in the CsPbI_3_ nanorods (Figure 3b–d). The results demonstrate that Cs (red), Pb (green), and I (blue) are uniformly distributed throughout the nanorods. The measured elemental stoichiometry ratio is 13.5:15.7:38.9, which is in excellent agreement with the theoretical ratio of 1:1:3. This confirms that the synthesized CsPbI_3_ nanorods possess good chemical homogeneity and stability. Furthermore, the EDS analysis clearly reveals that the distribution of phosphorus (P) overlaps significantly with that of Cs, Pb, and I, indicating that TOP is uniformly distributed on the surface of the nanorods. This provides direct evidence of its role in surface modification during the nanorod synthesis process.

The XRD pattern shown in Figure 3f further reveals the crystallographic features of the sample. All of the diffraction peaks align precisely with the peak positions of the standard α-phase CsPbI_3_ (PDF#97-018-1288), confirming the phase purity of the synthesized product. Notably, the XRD pattern exhibits two prominent diffraction peaks around 2θ ≈ 14° and 2θ ≈ 29°, which correspond to the (001) and (002) crystal planes of CsPbI_3_ perovskite, respectively. This indicates that the nanorod assembly is composed of highly crystalline α-phase CsPbI_3_ perovskite and also reflects the preferential orientation of the sample along specific crystallographic directions. These results further validate that the synthesized CsPbI_3_ nanorods possess excellent crystal quality and a well-defined phase structure.

**Figure 4:**
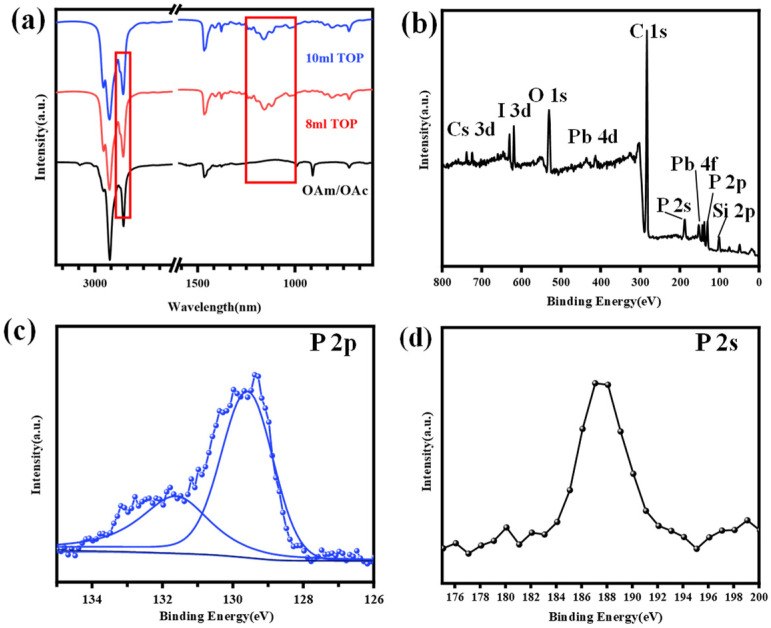
(**a**) FTIR spectra of TOP-CsPbI_3_. (**b**–**d**) XPS spectra of TOP-CsPbI_3_. (**b**) Survey, (**c**) P 2p, and (**d**) P 2s elements for the CsPbI_3_ nanorods.

Additionally, we characterized the ligands on the surface of the nanorods using Fourier-transform infrared (FTIR) spectroscopy. As shown in Figure 4a, compared to the traditional synthesis method using OA and OAm as ligands, the nanorods synthesized with TOP as the solvent exhibit a small, broad feature peak at 1155 cm^−1^, corresponding to the stretching vibration of the C-P bond in TOP [33,34,35]. Moreover, a stretching vibration peak of the CH_2_ bond in TOP is observed at 2876 cm^−1^. The precise position and intensity variations in these characteristic peaks suggest that a specific chemical interaction may occur between the TOP and CsPbI_3_ nanorods. This result further demonstrates that TOP, in addition to serving as a solvent during the nanorod synthesis process, may also participate in surface modification through coordination, thereby influencing the structure and stability of the nanorods.

Additionally, we analyzed the surface chemical states of the CsPbI_3_ nanorods (CsPbI_3_-NRs) using X-ray photoelectron spectroscopy (XPS). As shown in Figure 4b–d and Figure S3, the XPS spectra of the TOP-treated CsPbI_3_ nanorods clearly display characteristic binding energy peaks corresponding to the primary elements of the perovskite nanorods (Cs, Pb, and I). Furthermore, weak characteristic peaks for phosphorus are observed in the binding energy ranges of 129–133 eV (P 2p_3/2_) and 189 eV (P 2s), indicating that TOP may exist in some form on the surface of the nanorods. Specifically, the I 3d orbital spectra show binding energy peaks for I 3d_5/2_ and I 3d_3/2_ at approximately 619 eV and 631 eV, respectively. In the Pb 4f orbital spectra, the binding energy peaks for Pb 4f_7/2_ and Pb 4f_5/2_ are located around 139 eV and 144 eV, respectively. These features are consistent with the chemical states of Pb^2^⁺ and I^−^ [36]. Comparison with the literature data shows that the binding energy positions for Cs, Pb, and I in the CsPbI_3_ nanorods align with previously reported values, which are all within the experimental error range. These results suggest that the CsPbI_3_ nanorods treated with TOP exhibit good stability in surface chemical states, and that TOP may influence the surface properties of the nanorods through surface modification.

**Figure 5:**
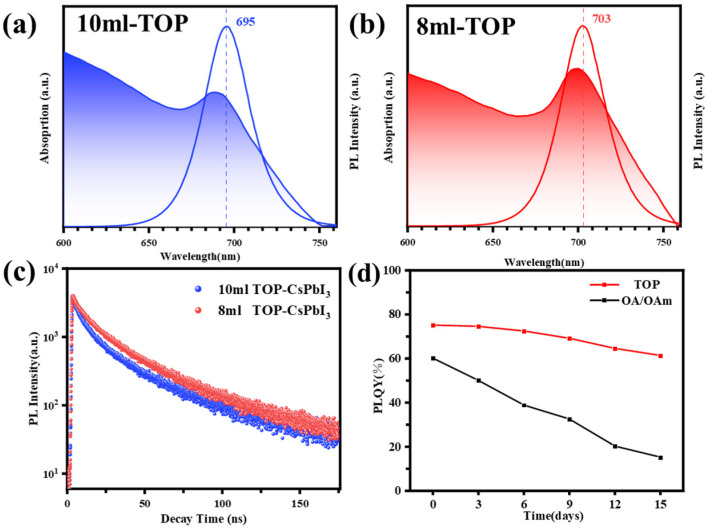
(**a**,**b**) PL and UV–vis absorption spectra of the CsPbI_3_ nanorods synthesized at 10 mL of TOP and 8 mL of TOP. (**c**) Time-resolved PL decay curves of the CsPbI_3_ nanorods. (**d**) Stability changes in the CsPbI_3_ perovskite PLQY synthesized in different systems.

Subsequently, we systematically characterized the optical properties of the CsPbI_3_ NRs using UV–visible absorption spectroscopy (UV-vis), photoluminescence spectroscopy, the photoluminescence quantum yield, and the fluorescence lifetime. As shown in Figure 5a,b, for the CsPbI_3_ nanorods treated with 10 mL of TOP (10 mL TOP-CsPbI_3_ NRs), the absorption peak is located at 690 nm, and the photoluminescence peak is at 695 nm. In contrast, for the CsPbI_3_ nanorods treated with 8 mL of TOP (8 mL TOP-CsPbI_3_ NRs), the absorption and photoluminescence peaks are located at 697 nm and 703 nm, respectively. As the TOP concentration decreases, the PL emission peak of the 8 mL TOP-CsPbI_3_ NRs exhibits a 13 nm red shift. This red shift can be attributed to the quantum confinement effect: the diameter of the 10 mL TOP-CsPbI_3_ NRs is approximately 9.6 nm, with some nanorods having diameters smaller than 9 nm, which is close to the Bohr radius (7 nm) of CsPbI_3_ perovskite. This leads to an enhanced quantum confinement effect, causing a blue shift in the fluorescence peak of the nanorods [37].

Additionally, we analyzed the time-resolved fluorescence spectra of the CsPbI_3_ nanorods using a double-exponential fitting method (Figure 5c). The results show that the average fluorescence lifetime (Γ_ave_) of the 10 mL TOP-CsPbI_3_ NRs is approximately 23.95 ns, while that of the 8 mL TOP-CsPbI_3_ NRs is slightly longer, around 27.86 ns. The PL lifetime data Γ_ave_ we provide consist of a short-lived component (Γ_1_) and a long-lived component (Γ_2_) [38,39,40]. The short-lived component is primarily associated with radiative recombination, while the long-lived component is related to non-radiative recombination. A_1_ and A_2_ represent the contributions of the radiative and non-radiative recombination processes to the overall recombination process, respectively. Detailed data can be found in Table S1. Both types of nanorods we synthesized have a dominant A_1_ parameter, which indicates that the surface passivation is effective, with fewer surface defects. As a result, radiative recombination dominates, leading to a high PLQY. As shown in Figure 5d, compared to the CsPbI_3_ perovskite synthesized using traditional ligand methods, the 10 mL TOP-CsPbI_3_ NRs exhibit outstanding photoluminescence performance, with an initial PLQY as high as 80%, and, after being stored in n-hexane solution at room temperature with 50% relative humidity for two weeks, it was able to retain 90% of its initial luminescent efficiency. This result demonstrates that the CsPbI_3_ nanorods treated with TOP not only exhibit a significant improvement in photoluminescence efficiency but also show excellent long-term stability, laying a solid foundation for the practical application of cesium lead iodide perovskite nanorods.

## 4. Conclusions

In summary, we have innovatively employed TOP as both the surface ligand and solvent to successfully synthesize α-CsPbI_3_ NRs with a high stability. This method enables the synthesis of size-controlled, monodisperse α-phase nanorods with lengths of 1 μm and 150 nm, and an aspect ratio of 10:1. These nanorods exhibit a high PLQY of approximately 80%, along with excellent environmental stability, as evidenced by a retention of 90% PLQY after 15 days of storage. Transient absorption spectroscopy reveals a carrier lifetime of up to 23.95 ns and 27.86 ns. Through the XPS and FTIR analyses, we investigated the adsorption and binding of TOP on the surface of cesium lead iodide perovskite. We present a simple, yet versatile, synthesis method for the production of highly stable α-CsPbI_3_ nanorods, significantly expanding their potential for a wide range of practical applications.

## Data Availability

The original contributions presented in this study are included in the article/Supplementary Materials. Further inquiries can be directed to the corresponding author.

## Supplementary Materials

The following supporting information can be downloaded at: https://www.mdpi.com/article/10.3390/ma18071518/s1. Figure S1: TEM images of CsPbI_3_ nanorods synthesized at 6 mL of TOP. Figure S2: The corresponding diameter and length distribution histograms of 6 mL TOP CsPbI_3_. Figure S3: XPS spectra of TOP-CsPbI_3_. Table S1. The detailed recombination lifetimes described by the double-exponential fitting method.
