# Supplementary material for: CsPbI3 Perovskite Nanorods: Enhancing Fluorescence Efficiency and Environmental Stability via Trioctylphosphine Ligand Coordination
Source: Materials (Basel). 2025 Mar 28;18(7):1518. doi: 10.3390/ma18071518 (PMC11990055; doi:10.3390/ma18071518)
Supplement: Supplementary file 1 [file materials-18-01518-s001.zip › materials-3535155-supplementary.pdf]

Supporting Information

$CsPbI_3$ Perovskite Nanorods: Enhancing Fluorescence Efficiency and Environmental Stability via Trioctylphosphine Ligand Coordination

Chengqi Liu†, Zahir Abdalla†, Xiaoqian Wang, Manrui Liu, Yanhui Jiao, Zisheng Tang, Qi Zhang and Yong Liu*

International School of Materials Science and Engineering (ISMSE), State Key Laboratory of Advanced Technology for Materials Synthesis and Processing, Wuhan University of Technology, Wuhan 430070, China;
liuchengqi42@163.com (C. L.); zahiralbashir3@gmail.com (Z.A.); 303568@whut.edu.cn (X.W.); liumanr14@163.com (M. L); j1769877205@163.com (Y. J.); tangzs3076@163.com (Z. T.); zq13307239180@163.com (Q.Z.)
*Correspondence:liuyong3837@whut.edu.cn
† These authors contributed equally to this work.

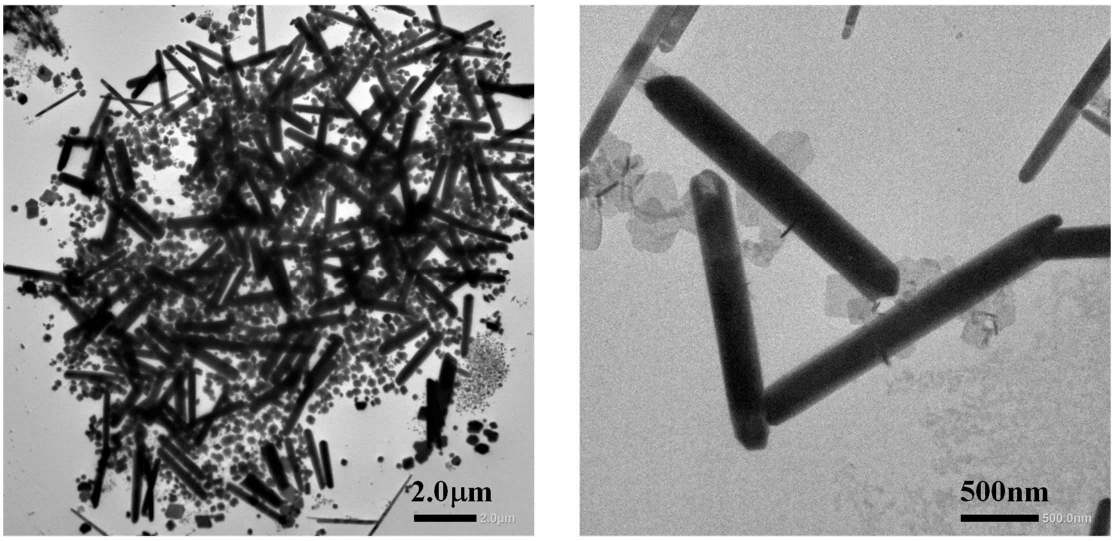

Figure S1. TEM images of $CsPbI_3$ nanorods synthesized at 6ml of TOP.

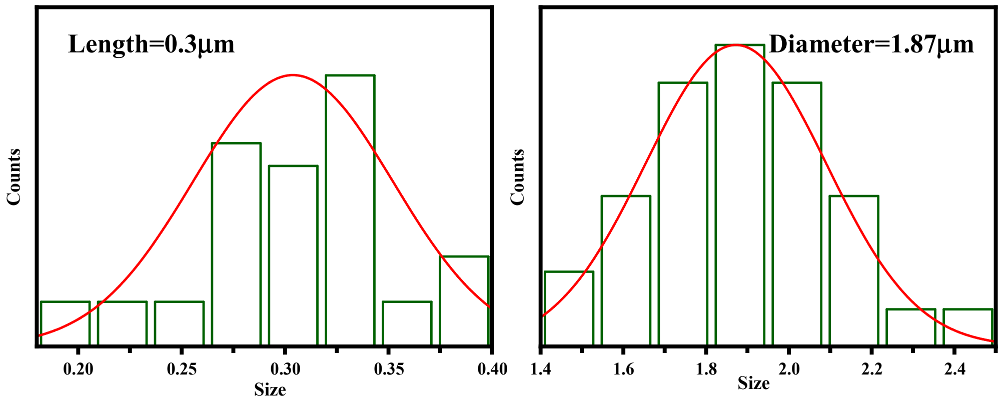

Figure S2. The corresponding diameter and length distribution histograms of the 6ml TOP $CsPbI_3$.

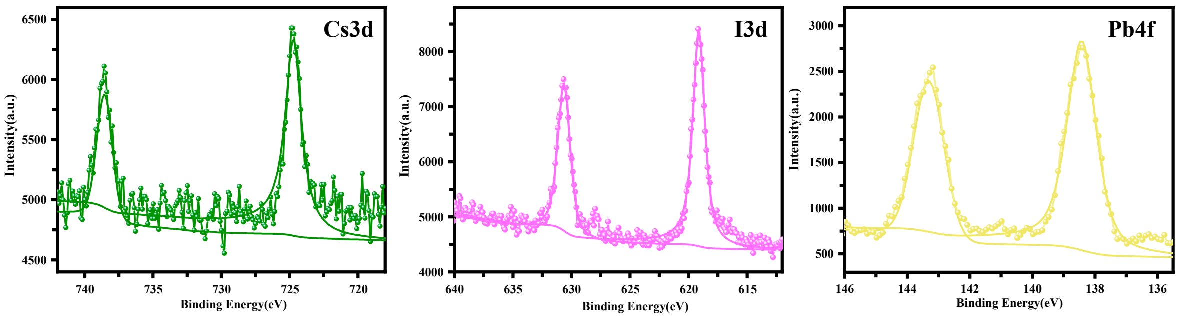

Figure S3. XPS spectra of TOP-$CsPbI_3$.

Table S1. The detailed recombination lifetimes described by the double-exponential fitting method.

Sample	Γ_1 (ns)	A_1(%)	Γ_2 (ns)	A_2(%)	Γ_{ave} (ns)
**10ml-TOP-$CsPbI_3$**	6.17	64.9	30.57	35.1	23.95
**8ml-TOP-$CsPbI_3$**	8.22	55.6	33.82	44.4	27.86

The fitting formula and calculations are as follows:

$$Y = A_1 exp\left({-t}/{\Gamma_1}\right) + A_2 exp\left({-t}/{\Gamma_2}\right)$$

$$\Gamma_{ave} = \sum A_i \Gamma_i^2 / \sum A_i \Gamma_i$$

where Γ_{ave} consists of a short-lived component (Γ_1) and a long-lived component (Γ_2). The short-lived component is primarily associated with radiative recombination, while the long-lived component is related to non-radiative recombination. A_1 and A_2 represent the contributions of radiative and non-radiative recombination processes to the overall recombination process.
